# Understanding neuropsychiatric symptoms in Alzheimer’s disease: challenges and advances in diagnosis and treatment

**DOI:** 10.3389/fnins.2023.1263771

**Published:** 2023-09-05

**Authors:** Andrew Pless, Destany Ware, Shalini Saggu, Hasibur Rehman, John Morgan, Qin Wang

**Affiliations:** ^1^Department of Neuroscience and Regenerative Medicine, Medical College of Georgia at Augusta University, Augusta, GA, United States; ^2^Department of Neurology, Medical College of Georgia at Augusta University, Augusta, GA, United States

**Keywords:** Alzheimer’s disease, neuropsychiatric symptoms, comorbidity, pharmacotherapy, clinical diagnosis

## Abstract

Neuropsychiatric symptoms (NPS) in Alzheimer’s disease (AD) affect up to 97% of AD patients, with an estimated 80% of current AD patients experiencing these symptoms. Common AD-associated NPS include depression, anxiety, agitation, aggression, and apathy. The severity of NPS in AD is typically linked to the disease’s progression and the extent of cognitive decline. Additionally, these symptoms are responsible for a significant increase in morbidity, mortality, caregiver burden, earlier nursing home placement, and greater healthcare expenditure. Despite their high prevalence and significant impact, there is a notable lack of clinical research on NPS in AD. In this article, we explore and analyze the prevalence, symptom manifestations, challenges in diagnosis, and treatment options of NPS associated with AD. Our literature review reveals that distinguishing and accurately diagnosing the NPS associated with AD remains a challenging task in clinical settings. It is often difficult to discern whether NPS are secondary to pathophysiological changes from AD or are comorbid psychiatric conditions. Furthermore, the availability of effective pharmaceutical interventions, as well as non-pharmacotherapies for NPS in AD, remains limited. By highlighting the advance and challenges in diagnosis and treatment of AD-associated NPS, we aspire to offer new insights into the complexity of identifying and treating these symptoms within the context of AD, and contribute to a deeper understanding of the multifaceted nature of NPS in AD.

## Graphical Abstract

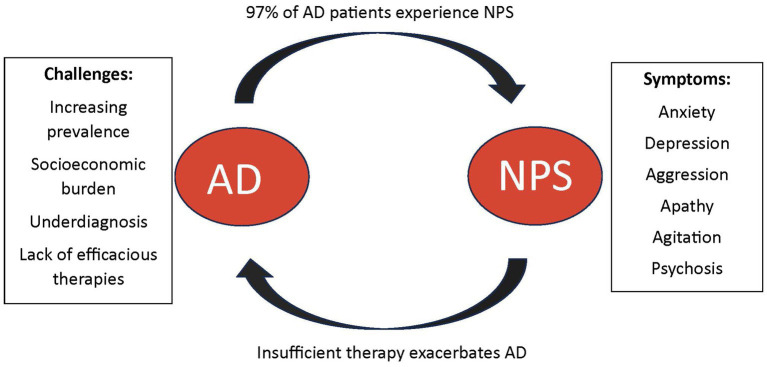

## 1. Introduction

Alzheimer’s disease (AD) is the most common neurodegenerative disorder in the realm of aging and cognitive impairment, the sixth leading cause of death among all adults in the United States, and the fifth leading cause of death in adults over the age of 65 worldwide (McDonald, 2017; Wong, 2020). As of 2023, about 1 in 9 individuals in the U.S. over the age of 65 have been diagnosed with AD, resulting in an estimated 6.7 million Americans over the age of 65 currently diagnosed (Rajan et al., 2021). Prevalence of earlier onset dementia is estimated at rates of 110 per 100,000 individuals aged between 30–64 (Hendriks et al., 2021). In 2018, AD claimed the lives of more than 122,000 individuals, which was a 146% increase from the year 2000 (Wong, 2020). The most recent U.S. census states there are 56 million persons over the age of 65; by 2030, it is projected that this value will be over 73.1 million (Bureau USC, 2023). This equates to a surge of 65% growth in our population of people over the age of 65 in less than a decade. The prevalence of AD is estimated to triple over the next 40 years due to both longer life expectancies and demographic changes (Barnes and Yaffe, 2011). Additionally, AD is more commonly seen in women than men, with a relative risk of 2.0 over men (Podcasy and Epperson, 2016). These differences in sex have been reported in literature to be contributed to many potential factors including but not limited to biological processes differences, consistency in health check-ups, and the life-expectancy disparities that currently exist (Mielke, 2018). Women on average live longer than men and thus are more likely to experience cognitive decline (Ginter and Simko, 2013).

Neuropsychiatric Symptoms (NPS) and disorders are frequently observed in AD, affecting up to 97% of all AD patients during their illness, which places significant burden on their caregivers (Bureau USC, 2023). The presence of NPS in AD is typical of more severe cognitive decline and advancing disease and is associated with increased morbidity and mortality (Clement et al., 2020). The cost of NPS in AD can range from $15,000–$85,000 per patient each year, exacerbating the socioeconomic burden of this disease (Cloutier et al., 2019). With the increasing prevalence of AD, AD-related expenditures are expected to rise from $305 billion in 2020 to $1.1 trillion by 2050 (Wong, 2020). Additionally, a 2019 survey estimated the burden that is upon caregivers who help with these costs or use it to hire formal care to be quite significant; there are approximately 16.3 million informal caregivers of patients with AD provided an estimated 18.6 billion hours of unpaid care (Wong, 2020). AD patients with NPS are more likely to be institutionalized, increasing the financial burden to nearly $4.3 billion dollars annually; nearly $85,000 per patient (Cloutier et al., 2019). These values do not take into consideration comorbidities that may be present. Numerous studies outline the financial burden that AD places on patients and their families (Aschenbrenner et al., 2023; Brockmann et al., 2023; Mahase, 2023).

The increasing prevalence of AD and its substantial socioeconomic impact emphasize the need for further research, earlier diagnosis, and better therapeutics for the primary disease process as well as NPS associated with the disease. In clinical settings, distinguishing and accurately diagnosing NPS associated with AD remains a challenging task, and it is imperative to gain a better understanding of the diverse clinical presentations of these symptoms. Although NPS affects nearly all AD patients, the overall clinical research for pharmaceutical therapeutics remains scarce (Steinberg et al., 2008). Many pharmacotherapies in use today for NPS in AD pose many side-effects and long-term risks; often neglecting to alleviate overall symptoms of NPS. Some therapies in use have been shown to even progress cognitive impairment and disease overall; however there are no other options for these patients at times (Tampi et al., 2016).

This review will discuss the current research pertaining to clinical diagnosis, manifestation of NPS, and current pharmaceutical therapies that are available. With the recognition of the complexities of identifying and treating these symptoms within the context of AD, it is critical to acknowledge the need for further research to counter the increasing public health burden on our expanding AD population and their caregivers.

## 2. Diagnosis and challenges

AD is thought to have several pathophysiological and biochemical changes that cause progression of disease such as hyperphosphorylation of tau proteins, amyloid-beta cleavage, presence of specific genetic phenotypes, and other idiopathic causes (Yarns et al., 2022). Definitions have been established by five different groups for diagnoses of preclinical and clinical AD (NIA-AA criteria, Dubois Criteria, Winbald Criteria, International Working Group-2 criteria, and the NIA-AA Research Framework Criteria) (Cummings et al., 2020). AD has an insidious onset that progressively worsens in severity over many years. The progression rate and manifestation of symptoms vary among individuals, but there are many symptoms that are classically seen. The initial symptoms are usually more cognitive-based and include aspects of memory, mostly in forming new memories (Kumar et al., 2023). These symptoms are typically followed by deficits in visuospatial function, judgment, language, and attention (Tahami Monfared et al., 2022). As the disease progresses, there is increasing decline in executive function, as well as development of NPS such as psychosis, depression, anxiety, agitation, aggression, and apathy (Lyketsos et al., 2011). Once thought to primarily occur in only late-stage disease, many studies have shown emergence of these symptoms in many individuals quickly after diagnosis (Lyketsos et al., 2011). As progression continues, individuals become less able to perform basic activities of daily living (ADLs) and instrumental activities of daily living (IADLs) and become increasingly reliant on caregivers as they develop incontinence and express extrapyramidal symptoms (Tsolaki et al., 2001; Bartolone et al., 2021). Once diagnosed, AD will continue to increase in severity over a span of 3–10 years (Zanetti et al., 2009). The end stage of AD is a complete loss of cognition, disorientation, and loss of language eventually leading to a near vegetative state culminating with anorexia, cachexia, and death (Minaglia et al., 2019).

There are memory deficits that arise as a part of normal aging that may not only concern patients but also make a correct diagnosis more difficult. Patients will commonly seek advice from their healthcare provider with complaints of normal aging, which must be discerned by the provider from a true cognitive disorder. Aspects of normal aging include problems with recall of recent memories, lapses in attention and language retrieval, decreases in processing speed, reasoning, problem solving, and fluid intelligence (Lo, 2017). Many of these symptoms may overlap with cognitive disorders; therefore, further investigation must be conducted. For the diagnosis of AD to be made, other systemic, infectious, neurologic, psychiatric diseases, and medication side effects must be ruled out (Breijyeh and Karaman, 2020). These additional causes of cognitive decline also make the diagnosis of AD more difficult, especially if they are not thoroughly investigated resulting in misdiagnosis. Once other causes have successfully been ruled out, patients are evaluated in a clinical setting by taking careful history with the utilization of tools such as the Montreal Cognitive Assessment (MoCA) or the Mini-Mental State Examination (MMSE) to evaluate for cognitive impairment (Brown et al., 2016). The DSM-V criteria for AD is currently the diagnostic standard for diagnosing AD (Apostolova, 2016). With a supportive history, evidence of cognitive impairment, and criteria stated in DSM-V, individuals are often referred for neuropsychological testing to further categorize the type of dementia (unless too cognitively impaired for this to be enlightening), evaluate specific deficits in the individual, and identify areas of relative strength for that individual (Chapman et al., 2010). Additionally, CSF analysis may also be conducted, along with PET or SPECT imaging to further increase the probability of an accurate diagnosis being made (Valotassiou et al., 2018; McGrowder et al., 2021). Even with advanced diagnostic techniques such as CSF analysis and PET/SPECT imaging, AD remains a largely clinical diagnosis to date (Dubois et al., 2021). Table 1 summarizes the clinical presentation of NPS at differing stages of AD and current pharmacological interventions that are standardized today.

**Table 1:** Summary of clinical stages of AD and associated NPS and treatment in comparison to MCI.

Clinical stage	MCI	Mild AD	Moderate AD	Severe AD
MMSE score	26+	>20	10–19	<9
Average duration of disease stage	4–10 Years	2–4 Years	2–10 Years	1–3 Years
Behavioral/Psychological symptoms reported	Apathy, Anhedonia	Apathy, Depression, some Anxiety	Apathy, Delusions, Major Depression, Anxiety, Insomnia, Agitation, Comprehension, Aggression	Apathy, decreased Depression, IADLs affected, Psychosis, Aggression, Agitation, Comprehension, worsened Insomnia
Current pharmacological therapies	Acetylcholinesterase Inhibitors, Donepezil	Acetylcholinesterase Inhibitors, Methylphenidate, SSRIs/SNRIs, Antidepressants, Carbamazepine, Donepezil	SSRIs/SNRIs, Lecanemab, Brexpirpazole, Benzodiazepines, Atypical Antipsychotics, Antidepressants	Atypical Antipsychotics, Benzodiazepines, Antidepressants

While accepting a diagnosis of AD is difficult for both patients & caregivers alike, ensuring understanding of the disease manifestations is important. Complicating the clinical picture is the frequent presence of anosognosia (lack of awareness) for cognitive deficits present in many patients with AD (Salmon, 2010). As the disease progresses, the patient will become increasingly deficient in ADLs and will need progressive assistance from caregivers (Potkin, 2002). The emergence of NPS such as psychosis, agitation, aggression, anxiety, depression, and apathy have been shown to be associated with increased caregiver burden, increased institutionalization, as well as a more rapid cognitive decline in general (Clement et al., 2020).

In clinical practice, diagnosis of these NPS can be difficult to make in AD because it is not always transparent whether a behavior is a symptom of AD or part of a comorbid or preexisting psychiatric condition. For example, a patient may have a concomitant diagnosis of major depressive disorder alongside AD, or their depression may simply be the direct result of neurochemical changes from their AD. This can complicate proper diagnosis which is important for proper treatment. Psychosis and delusions may alternatively be mislabeled as an NPS as well when there may be an accompanying psychotic disorder, which may require different treatment. Unfortunately, there are not many well-defined diagnostic criteria for NPS specifically in AD making these diagnoses more difficult, other than recent criteria regarding psychosis and depression (Jeste and Finkel, 2000; Lyketsos et al., 2002).

The original criteria for diagnosing NPS in AD patients was first established by Jeste and Finkel in 2000 who described delusions and hallucinations (Jeste and Finkel, 2000). In this criterion, the psychosis that is prevalent during prodromal or MCI is not included as a reason for diagnosis. Years later, the International Society to Advance Alzheimer’s Research and Treatment (ISTAART) established their own criteria for diagnosing NPS in AD (Ismail et al., 2017). This criterion emphasizes the magnitude of a clear change from an individual’s typical behavior or personality that persists for at least 6 months. These changes may be in the domains of apathy, affective dysregulation, difficulties with impulse control, social inappropriateness, abnormal perception, and thought of content (Lussier et al., 2020). Therefore, it is important to screen patients by taking a thorough history or by using simple methods such as screening questionnaires such as the neuropsychiatric inventory (NPI) to recognize NPS symptoms early and begin treatment (Kaufer et al., 1998). While it is complicated to make proper NPS diagnosis in AD, it is imperative that further research and criteria be brought forth. Early diagnosis of NPS in AD has shown to significantly improve the quality of life for both the patient as well as caregivers (Eikelboom et al., 2019), especially with the associations of institutionalization and accelerated cognitive decline with NPS (Hongisto et al., 2018; Connors et al., 2020).

## 3. Clinical presentations and management of Alzheimer’s disease-associated neuropsychiatric symptoms

### 3.1. Depression

The DSM-V defines depression roughly as feelings of worthlessness or excessive or inappropriate guilt nearly every day, typically with loss of interest or pleasure in nearly all activities (American Psychiatric Association, 2013). Depression in AD is devastating for both the patient and the caregivers. For the patient, depression in AD has been shown to more significantly decrease the quality of life, cause greater impairment in ADLs, worsen cognitive function, increase the likelihood of physical aggression, and cause earlier nursing home admission (Lyketsos and Olin, 2002; Pfeifer et al., 2013; Brzezińska et al., 2020). Depression in this population has also shown an association with higher mortality and suicide rates (Petersen et al., 2017; Conejero et al., 2018). Interestingly, as AD advances, there is increasing caregiver burden leading to a higher prevalence of depression among caregivers, which in turn could affect the prognosis and quality of care in the dementia patient (Medrano et al., 2014; Huang, 2022). Nearly 80% of patients with AD have family members as their caregivers (Haley, 1997). Over half of AD patients need care for greater than 8 hours a day which can be both exhausting and frustrating (Fuh et al., 1999). Studies have shown that caregiver depression occurs in roughly 30–50% of cases, some studies showing even up to 83% of cases (García-Alberca et al., 2011; Sallim et al., 2015; Ying et al., 2019).

A meta-analysis of 63 studies conducted in 2015 showed that greater than 40% of individuals diagnosed with AD will experience significant depressive symptoms throughout the course of their diagnosis, compared to roughly 10–15% of the general population (Chi et al., 2015; Daly et al., 2021). Causes of depression in AD have been investigated although there are likely complex interactions between many factors (Brzezińska et al., 2020). There are many known psychological factors related to being diagnosed with AD including a sense of loss of control, stress with knowing cognitive and functional abilities will decline, and perceived guilt which will occur from anticipated caregiver burden and becoming less autonomous. Interestingly, there have been studies shown that up to 80% of patients in long term care facilities with AD experience some sort of pain, which is suspected to cause depression, agitation, and other symptoms in many cases (Scuteri et al., 2022). Some studies have shown that treatment of pain in some patients can even significantly reduce depression symptoms in AD (Husebo et al., 2014).

It has largely been shown that depression is linked to a decrease in serotonin levels in the brain, largely supported by a decrease in symptomatology with the use of serotonin increasing medications (Fuller, 1991; Delgado, 2000; Albert et al., 2012). There is also evidence that suggests depression in AD may be tied to other monoaminergic systems as well (Nutt, 2008). Selective serotonin reuptake inhibitors (SSRIs) remain the first-line treatment for depression in individuals with AD. The main choice of SSRIs for depression in AD is largely based off the fact that they tend to portray less side effects than other antidepressants, such as many atypical antidepressants, tricyclic antidepressants, serotonin and norepinephrine reuptake inhibitors (SNRIs), and monoamine oxidase inhibitors (MAOIs) (Santarsieri and Schwartz, 2015). While SSRIs have been the mainstay of treatment for depression in the general population, many recent studies have called into question their effectiveness for individuals with depression associated with AD. While some studies have shown their effectiveness along with other antidepressants in AD (Banerjee et al., 2011), other large-scale studies and meta-analyses have found no significant difference between SSRIs and placebo (Orgeta et al., 2017; Dudas et al., 2018). In addition, studies have interestingly shown a possible link between long-term antidepressant use and an increased risk of developing AD, as well as an increased risk of developing AD in depressed individuals (Ownby et al., 2006; Kessing et al., 2009; Wang et al., 2018). Cognitive decline has been observed with the use of certain SSRIs such as citalopram (Porsteinsson et al., 2014). Moreover, even depression has been suggested as a risk factor and catalyst for cognitive decline in the elderly, especially in the instance of AD (Lohman et al., 2013; Mdawar et al., 2020). This brings forth the need for further high-quality studies on the efficacy of SSRIs and other antidepressants to examine the true efficacy in AD, or other therapeutic interventions for depression in AD.

While pharmacotherapy is the mainstay in treatment for depression in AD, exercise and cognitive behavioral therapy has been shown in some studies to reduce depressive symptoms more than twice than standard medical therapy (Teri et al., 2003). Improvements of mood in caregivers has been shown to produce positive effects on NPS of the patients that they care for. Cognitive behavioral therapy (CBT) has also been shown to help with depression and other mood symptoms which thus may secondarily reduce the rates of depression in patients with AD (Gallagher-Thompson and Coon, 2007). Additionally, both cognitive stimulation and psychological therapies have been shown to reduce rates of depression in AD (Orgeta et al., 2015; Fukushima et al., 2016).

### 3.2. Anxiety

The DSM-V defines anxiety as an excessive worry and apprehensive expectations which occur more than most days in a week, about a number of events (DSM-V). However, there is no subsequent definition for anxiety related to AD or to other dementias. Anxiety affects both the patient and the caregiver by exacerbating the burden of disease. For the patient, anxiety has been shown to negatively affect memory, attention, and cognitive abilities. Once established, anxiety in AD has been shown to specifically worsen cognitive decline, lead to premature institutionalization, decrease quality of life, increase caregiver distress, and lead to increased hospital visits with subsequent increased cost of care (Finkel, 2000; Riley et al., 2014; Mendez, 2021). Upon diagnosis, patients with AD may experience a fear of the unknown future, as well as feelings of insecurity (Cox, 2007). This may cause patients to become hyperalert and misinterpret normal stimuli as dangerous (Boyd, 2008).

Anxiety disorders are classified as among the most common psychiatric disorders in both the general population as well as the AD population (Lahousen and Kapfhammer, 2018; Mendez, 2021). A 2021 meta-analysis of 48 studies showed a pooled prevalence for anxiety in AD reaches up to 39% of patients (Mendez, 2021). In 2007, Starkstein et al. proposed diagnostic criteria for generalized anxiety disorder in AD, which were based on the DSM-IV and the International Classification of Diseases, 10th (World Health Organization, 2004). These criteria encompassed symptoms such as irritability, muscle tension, restlessness, fears, and respiratory distress, occurring within the context of anxiety and worry (Starkstein et al., 2007).

Interestingly, anxiety has also been shown to be associated with an increased risk for developing dementia and cognitive impairment (Gulpers et al., 2016; Santabárbara et al., 2020). Gulpers et al. (2016) conducted a meta-analysis in 2014 showing a 57% higher risk of developing dementia in older adults if anxiety was present. The study also showed that the association was higher with increased age, suggesting that anxiety could be an early symptom of dementia. Anxiety has also been shown to increase the overall rates of conversion from MCI to dementia (Somme et al., 2013; Stella et al., 2014; Mah et al., 2015; Li and Li, 2018).

Initial management for anxiety in AD includes conservative management using cognitive stimulation, reassurance and redirection, social support, maintaining a calm environment, and minimizing anxiety-evoking triggers (Mendez, 2021). Group music interventions have also been shown to reduce dementia-associated anxiety (Ing-Randolph et al., 2015). A 2019 systematic review shows that cognitive stimulation therapy, music-based therapies and interventions, as well as CBT were the only 3 nonpharmacological interventions for anxiety and depressive psychiatric that improved cognition (Kishita and Laidlaw, 2017). Routine chores, or other repetitive tasks to stay busy may help decrease the anxiety in a patient with AD as they do not have to make decisions or remember what they should be doing. Physical exercise has also been suggested to modulate amyloid turnover and overall reduce anxiety in AD (De la Rosa et al., 2020).

If a patient’s anxiety is not amendable to conservative management, there are few pharmacotherapeutics that can be trialed. For the acute management of anxiety, benzodiazepines and antipsychotics have historically been used for treatment (Tampi et al., 2016; Mendez, 2021; Joyce et al., 2022). This is problematic as benzodiazepines are well known to be dangerous when used in the elder population, and antipsychotics even have a black box warning in use of AD (Steinberg and Lyketsos, 2012; Gress et al., 2020). For a more chronic approach of anxiety, SSRI/SNRIs and buspirone are the mainstays of treatment.

Pharmacologic therapies for anxiety related symptoms typically default to 5-HT anti-depressant class. A systematic review published in March 2023 studied the efficacy of commonly used drugs to treat anxiety for anxiety in AD in randomized clinical trials against placebos. These studies showed that the efficacy using mirtazapine, sertraline, and trazodone did not result in a statistically significant difference when compared to the placebo (SMD = −0.08; 0.31; and 0.03, respectively) (Chen et al., 2023). However, 5-HT anti-depressants such as Citalopram showed an efficacy of 94.8% (SMD = −0.44, 95% CI = −0.72 to −0.16) when compared to placebo and other anti-depressant drugs (Albert et al., 2012). Although the efficacy for some of these anti-depressants shows promise for therapy, several known side-effects are reported for these anti-depressants including, nausea, drowsiness, GI upset, xerostomia, headache, appetite changes, etc. (Delgado, 2000). Although patients that experience anxiety in AD struggle to find a pharmacologic therapy that will sustain symptoms as well as minimize side-effects, there are no current alternative options for them. Thus, most patients try to ignore the side-effects of the medication in order to control their anxiety related symptoms.

### 3.3. Psychosis

Psychosis refers to a set of symptoms consisting of delusions, hallucinations, and disorganized thought and speech (Richards and Gurr, 2000). Psychosis in AD patients is one of the most clinically relevant NPS in AD and may be one of the most difficult to control. Psychosis in AD is associated with the concurrence of other behavioral disturbances, and the utmost bothersome are agitation and aggression (Schneider et al., 2003; Ismail et al., 2022). Furthermore, psychosis in AD is correlated with accelerated cognitive decline, increased mortality, increased rates of institutionalization, increased cognitive impairment, as well as higher distress for family and caregivers (Sweet et al., 2003; Balieiro et al., 2010; Sinha et al., 2017).

According to the DSM-V, the diagnosis of psychosis in AD is labeled as, “major neurocognitive disorder due to AD with a behavioral disturbance” (Gaebel and Zielasek, 2015; Cummings et al., 2020). Psychosis mostly affects individuals in late-stage disease but has also been shown to affect some patients in early disease (Ismail et al., 2022). It is estimated that up to 50% of AD patients will experience some features of psychosis throughout their diagnosis, although the severity and duration of psychosis vary among individuals (Murray et al., 2014). There is likely an underdiagnosis of psychosis in AD due to the frequent misdiagnosis of Lewy Body Dementia (LBD)—which differs in treatment from AD (Ismail et al., 2022). In AD, common delusions include delusions of theft, infidelity, persecution, abandonment, identity, and location (Hwang et al., 1997; Reeves et al., 2012; Hashimoto et al., 2015). In AD, hallucinations can occur in any sensory modality, but most common are visual hallucinations (Holroyd and Sheldon-Keller, 1995; Pezzoli et al., 2022). Hallucinations are mostly seen in later stage disease (El Haj et al., 2017). Aphasia and psychosis may cause disorganized thought and speech which may manifest as inability to organize thoughts to produce proper speech, as well as difficulties understanding others (Woodward, 2013). This may increase confusion and lead to other symptoms seen in AD such as frustration and agitation. While this is seen early in disease, it significantly exacerbates difficulties with social function as it progresses.

If psychosis occurs in an AD patient it is important to evaluate for offending medications (e.g., anticholinergics and antihistamines) and to rule out urinary tract infections, pneumonia, or other infections that may cause delirium on top of their existing AD. In the case of psychosis, the safest initial treatment modality is reorientation with communication and environmental simplification. The symptoms described above can lead to multiple bouts of acute onset confusion and disorientation throughout the day, and the most easily tolerated modality is redirection and reorientation (Ismail et al., 2022). Keeping the environment simplistic for individuals with AD may help decrease rates of confusion and disorientation (Woodbridge et al., 2018). When AD individuals are taken out of known environments (e.g., admitted to the hospital) they may experience increased rates of disorientation. Keeping environments simple helps reduce sensory input in these individuals and keeping familiar objects around and in plain sight have been shown to help reduce disorientation (Davis and Weisbeck, 2016). This may become frustrating to caregivers as they must reorient patients several times a day, often repeating the same prompts repeatedly. There has been little research in using CBT for psychosis specifically in AD, although a very small series has suggested some benefit for its use in AD (Holroyd and Sheldon-Keller, 1995).

The main pharmacologic modality for treating psychosis in the general population is with typical and atypical antipsychotics. However, the use of these medications in the AD population is highly controversial and current research shows it should be an option of last resort. While severe psychosis and delusions can be potentially harmful for both the patient and the caregiver and may need to be eminently dealt with using antipsychotics, they may have many downsides in AD patients. First, these medications carry many side effects, including increased sedation and cognitive impairment which may exacerbate confusion (Ellul et al., 2007; Lopez et al., 2013; Calsolaro et al., 2019). They are also associated with extrapyramidal side effects, adverse cardiovascular effects including increased risk of stroke and myocardial infarction, as well as increased mortality overall (Blair and Dauner, 1992; Maust et al., 2015; Sahlberg et al., 2015; Nielsen et al., 2017). There is also evidence that use of antipsychotics in AD may hasten disease progression and should thus only be used as last resort medications when the risk of benefit outweighs the potential harm (Ellul et al., 2007).

### 3.4. Agitation and aggression

Although the exact definition varies from study to study, agitation and aggression in AD patients can resemble restlessness, excessive fidgeting, verbal abuses, shouting, as well as more violent physical activities such as hitting and throwing objects at the caregiver (Ballard and Corbett, 2013; Koenig et al., 2016). There are many suspected causes of agitation and aggression in AD, many related to basic needs that cannot be communicated well such as thirst, hunger, and pain. As discussed above, it may be stressful to constantly not be oriented to your location, your surroundings, your peers, and not know how to effectively communicate your wants and needs. Agitation may secondarily lead to verbal and/or physical aggression, resulting in harm to either the patient or caregiver. Similar to the other NPS, agitation and aggression in AD have been linked to increased cost of care, increased morbidity and mortality, decreased quality of life, increased rates of institutionalization, and increased caregiver burden (Gallagher and Herrmann, 2015).

Aggressive behaviors are reported to occur in approximately 30% of patients with AD throughout their disease, and possibly up to 60% among AD patients living in long term care facilities (Margallo-Lana et al., 2001; Lyketsos et al., 2002; Zuidema et al., 2007; Kverno et al., 2008). Those in long term care facilities are likely at later stages of AD as well as are likely placed in these homes due to worsening symptoms, hence the marked increase in prevalence.

Redirection and reorientation methods are often attempted after the onset of aggression and agitation (Carrarini et al., 2021). A common method for combating agitation and aggression in AD is to identify triggers in an attempt to avoid them. Caregivers should make sure the environment is safe and clear of dangerous objects and should always approach an agitated patient slowly using calm mannerisms. Recent students have shown that music therapy may result in calming agitated patients (Pedersen et al., 2017). Brief psychosocial therapy (BPST) has been used to treat agitation in AD patients. The CALM-AD trial showed that BPST improved 43% of the patient’s symptoms by 30% (Ballard et al., 2009).

Nonpharmacologic measures for treating agitation and aggression in AD are not always efficacious resulting in the need of pharmacologic measures. Antipsychotics, as discussed above, should be used carefully in AD due to their harmful side effects and potential for exacerbating the disease. Some studies even show that once treated with antipsychotics, withdrawal of these interventions can lead to worsening of behaviors such as agitation and aggression, initiating an unwanted feedback mechanism (Ballard et al., 2016). Benzodiazepines are also commonly tried in efforts to calm patients, but again there are many risks to these medications especially in the elderly (Gress et al., 2020). They have not only been shown to increase falls, but also have been shown to exacerbate symptoms such as confusion, delirium, and even accelerate cognitive decline (Markota et al., 2016; Picton et al., 2018). These medications may exacerbate agitation and aggression in certain individuals and will also likely need to steadily increase dosages as tolerance takes place, further increasing the risk of adverse effects (Defrancesco et al., 2015).

Mood stabilizers such as valproic acid have been used for decades to mitigate agitation and aggression symptoms; however, many recent studies have called its efficacy into question (Tariot et al., 2005; Lonergan and Luxenberg, 2009; Tariot et al., 2011). Along with a black box warning for hepatotoxicity, valproic acid has many other associated adverse effects such as GI upset, thrombocytopenia, coagulopathies, metabolic disorders, and even worsening cognitive dysfunction (Safdar and Ismail, 2023). This evidence is not in favor of using valproic acid for agitation and aggression in dementia despite having been used for such a long time (Gallagher and Herrmann, 2014; Baillon et al., 2018). Although some research has shown potential neuroprotective effects of valproic acid and that it may help mitigate rate of progression of AD (Zhang et al., 2010), recent studies have shown no difference in Mini-Mental Status Exam (MMSE) comparing valproic acid and placebo (Liu and Wang, 2020).

Carbamazepine has shown some efficacy in treating agitation and aggression, but there are significant side effects associated with this medication, mainly agranulocytosis which can be fatal if not carefully monitored (Lemke, 1995). There have been studies evaluating the efficacy of gabapentin, lamotrigine, and levetiracetam, but there is currently not enough evidence to support regular use in AD (Rayfield et al., 2014). Some providers may choose SSRIs, notably citalopram in attempt to mitigate these symptoms. SSRIs have been shown to significantly improve agitation and aggression after using these drugs (Porsteinsson et al., 2014); however, these drugs take 4–9 weeks to take full affect so cannot be used in the immediate phase. These drugs tend to have less side effects than the other ones mentioned, but may increase the risk of falls, agitation, and cardiac arrhythmias, and even worsen cognitive function in some patients (Porsteinsson et al., 2014).

As of May 2023, brexpiprazole (an atypical antipsychotic) was approved by the FDA specifically to treat agitation symptoms in AD. In recent Phase 3 clinical trials of a double-blind, placebo-controlled trial conducted by Otsuka and Lundbeck, there was a statistically significant difference (*p* = 0.0026) in the average mean change in baseline over 12 weeks using in the Cohen Mansfield Agitation Inventory with brexpiprazole versus placebo (Grossberg et al., 2023). The exact mechanism is unknown but brexpiprazole is suspected to be a partial agonist for both serotonin 5-HT1A and dopamine D2 receptors, as well as an antagonist at serotonin 5-HT2A receptors (Eaves and Rey, 2016). In the phase 3 clinical trials, the drug was generally well-tolerated and safe, compared to the other atypical antipsychotics when used in treatment of AD (Grossberg et al., 2023). More individuals in the treatment group died, although the deaths were ultimately decided to be unrelated to the medication.

### 3.5. Apathy

Apathy is defined as loss of motivation in at least two of three domains—emotion, goal directed behavior, or cognitive activity (Nobis and Husain, 2018). Patients with apathy demand management and support as well as increase caregiver burden and service utilization, as patients with apathy may not be able to make their own decisions, initiate actions, or work toward even life-sustaining goals (Chang et al., 2021). Apathy has been associated with increased rates of placement in long-term care facilities as well as with increased rates of cognitive decline (Richard et al., 2012; Breitve et al., 2018). In some studies, apathy has been shown to be more likely to facilitate the progression to AD or Lewy body dementia in patients with MCI (Breitve et al., 2018). In a population-based study, the presence of apathy was linked with 3 times higher mortality than those without apathy (Padala et al., 2020a).

Apathy is the most common NPS in AD which can arise early and remain throughout the progression in AD with prevalence ranging from 53–92% (Drijgers et al., 2009). It has been identified as one of the behavioral signs of executive cognitive dysfunction, which involves the selection, initiation, direction, implementation, and regulation of other cognitive skills and behavior (Ready et al., 2003). Closely associated, there has been a loss of empathy identified in many individuals diagnosed with AD (Fischer et al., 2019; Ávila-Villanueva et al., 2021). This can increase the burden on caregivers, as patients may not care or understand how their actions toward others may affect them. Apathy in AD may be difficult to detect, as patients do not directly express their condition, and thus must be recognized by either the caregiver or physician as abnormal. Given little motivation to do much of anything, apathy sufferers may often be mislabeled as having depression.

Before resorting to pharmacotherapy for treatment of apathy in AD, conservative management should be trialed. Simple cognitive stimulation, music therapy, art therapy, and even exercise have all been shown to decrease symptoms of apathy in AD (Buettner et al., 2011; Teixeira et al., 2021). Lastly, some studies have shown that the use of neuromodulation is both safe and may improve apathy and cognition in AD (Padala et al., 2020a).

While current evidence shows that acetylcholinesterase inhibitors and NMDA receptor antagonists may moderately improve NPS symptoms, the evidence for these medications in treating apathy is controversial. Some studies found no improvement compared to placebo (Kobayashi et al., 2016; Kishi et al., 2017), while another found only a slight beneficial effect compared to placebo (Rea et al., 2014). However, larger studies have shown that cholinesterase inhibitors may be the best treatment currently available that we have for apathy (Berman et al., 2012; Theleritis et al., 2018). *Ginkgo biloba* as well was found to be potentially helpful in reducing apathy in AD, but did not have significant cognitive benefits in randomized controlled clinical trials (Scripnikov et al., 2007; Bachinskaya et al., 2011). Methylphenidate, a psychostimulant, has shown some beneficial effect in improvement of apathy, with side effects of weight loss and increased anxiety (Rosenberg et al., 2013; Padala et al., 2020a). Modafinil and SSRIs have also been used in the past to try to improve apathy, although studies have shown no improvement in apathy or ADLs with modafinil (Frakey et al., 2012). One study even showed that SSRIs may be associated with an apathy-induced syndrome (Padala et al., 2020b). Antipsychotics have been shown to potentially increase apathy symptoms in AD patients, and as listed above, have considerable side effects in the AD population (De Deyn et al., 2004; Sultzer et al., 2008).

## 4. Discussion

AD affects patients, caregivers, and the overall public whether economically, socially, or through accessibility and is expected to surge in prevalence by 65% in the next decade (Steinberg et al., 2008; Nichols et al., 2022). NPS in AD is known to affect over 97% of AD patients with clinical symptoms of depression, anxiety, psychosis, agitation, aggression, and apathy, yet is lacking in conversation and research. Considering the current $305 billion AD related expenditures, the public health need for further research and discussion of NPS in AD patients is enormous (Cloutier et al., 2019). Currently, there is still not a standard-defined diagnostic criteria for all NPS in AD.

While there is no cure for AD, there are several classes of medications that aim to improve quality of life and functional capacity, as well as to improve psychiatric symptoms as they may arise. While the medications that are being used may temporarily suppress symptoms, they do not address the underlying pathology. In addition, many of these drugs have been shown to increase mortality, cognitive decline, and adverse events in AD. Although these therapies have been used for decades, there is a lot of controversies whether these drugs actually provide enough benefit to be cost-effective (Casey et al., 2010). There are several classes of drugs approved by the FDA for the treatment of AD, all of which may slightly improve rate of progression and symptomatology, but do not treat, reverse, or halt the progression of the disease. The drug classes currently approved include acetylcholinesterase inhibitors (Donepezil, Rivastigmine, and Galantamine), NMDA receptor antagonists (Memantine), and monoclonal antibodies (Aducanumab and Lecanemab) (Yiannopoulou and Papageorgiou, 2020; Haddad et al., 2022; van Dyck et al., 2022). The most recent class of drugs being researched are monoclonal antibodies (Aducanumab and Lecanemab) targeted against amyloid plaques, which in research trials (Haddad et al., 2022; van Dyck et al., 2022), have shown success in reducing amyloid and phosphorylated tau in the brain. These drugs have demonstrated modest (25–30%), but statistically significant reduction in both cognitive decline and ADL function at 18 months relative to placebo (Beshir et al., 2022). Additionally, these monoclonal antibodies can cause significant inflammatory/edematous or hemorrhagic brain lesions in up to 40% of treated AD patients (Mimica and Presecki, 2009; Laroche et al., 2013; Ferreira et al., 2020; Fink et al., 2020; Imai et al., 2020).

There is some evidence that supports that using the medications listed above for AD to treat cognitive deficits may also improve NPS (Hwang et al., 2004; Wang et al., 2015). In practice, routine clinical assessment should be performed to monitor the emergence of specific symptoms and overall burden to the patient and caregiver. While the medications listed above may decrease the prevalence and incidence of NPS in AD, these drugs do not specifically target NPS symptoms once they have emerged. While there have been a few recent accelerated FDA approvals for Lecanemab in AD and Brexpirprazole for agitation in AD, larger scale studies should be conducted to better assess clinically significant improvement. Currently, there are no drugs approved specifically for NPS in AD (outside of Brexpirprazole for agitation in AD), thus healthcare professionals must resort to other therapeutic interventions as listed above in hopes of suppressing emerging symptoms. Nonpharmacologic interventions may be attempted but these are often unsuccessful (Kales et al., 2014). In the few instances where medications may help alleviate NPS in AD, there are typically accompanying significant side effects that may either worsen the disease process or result in increased morbidity. Thus, there must constantly be a risk–benefit analysis conducted when deciding whether to use current pharmacologic interventions to outweigh beneficence with nonmaleficence with the current treatment strategies that are currently employed.

Further understanding of the neuropathogenesis of NPS in AD should be elicited to develop more targeted therapeutic approaches in order to mitigate NPS in AD globally. It is important to note that NPS specifically in AD may differ in etiology and biology compared to NPS in the general population. For example, AD is associated with early degeneration of noradrenergic neurons (Gannon et al., 2015; Gannon and Wang, 2018), which play critical roles in regulating multiple aspects of cognition and behavior. Moreover, the current understanding of how AD and NPS interact to exacerbate each other is not fully elucidated. It is known that there is a noticeable interaction between the two. Further research is needed to address these knowledge gaps in order to develop effective pharmaceutical therapies for NPS patients in AD (Steinberg et al., 2008). Such therapies would aim to alleviate the economic burden and overall known side effects, thereby combating the growing population affected. Future research should seek to evaluate more psychosocial interventions combined with mild anti-psychotics for a combination therapy to alleviate, stop, and eventually prevent NPS from occurring in AD. Public health professionals and clinicians should work toward developing a standardized definition to aid in the diagnosis and lead-time for treatment of NPS in AD overall, as well as for each symptom.

## 5. Conclusion

With increasing numbers of patients with AD as well as patients with AD suffering with NPS, there is an increased need for research on pathophysiology, diagnostic criteria, and therapy. NPS in AD remains a public health crisis affecting both the patients and caregivers. Each NPS in AD are correlated to worsening cognitive decline, increased morbidity for both the patient and caregivers, increased rates of institutionalization, and increased costs to our healthcare system. With the current increasing aging population, the healthcare system will soon be severely strained resulting in massive economic burden. Current pharmacological therapies are mildly effective at best and present risks such as worsening symptoms and progressing disease rather than maintenance and prevention of symptomatic burden. Further research is needed to recognize and diagnose NPS in AD earlier as well as both nonpharmacologic and pharmacologic therapies that alleviate symptoms and diminish the risk of exacerbating symptoms.

## Author contributions

AP: Conceptualization, Formal analysis, Investigation, Writing – original draft. DW: Formal analysis, Investigation, Writing – original draft. SS: Conceptualization, Formal analysis, Investigation, Writing – original draft. HR: Investigation, Writing – original draft. JM: Validation, Writing – review & editing. QW: Conceptualization, Funding acquisition, Project administration, Resources, Supervision, Writing – review & editing.

## Funding

The author(s) declare financial support was received for the research, authorship, and/or publication of this article.

This work is supported by NIH grants AG056815, AG064664, and AG067729.

## Conflict of interest

JM has received compensation from Eisai and Biogen as a consultant and Biogen as a speaker on AD disease state. QW serves as a member of the Scientific Advisory Board for Terran Biosciences.

The remaining authors declare that the research was conducted in the absence of any commercial or financial relationships that could be construed as a potential conflict of interest.

## Publisher’s note

All claims expressed in this article are solely those of the authors and do not necessarily represent those of their affiliated organizations, or those of the publisher, the editors and the reviewers. Any product that may be evaluated in this article, or claim that may be made by its manufacturer, is not guaranteed or endorsed by the publisher.

## Figures and Tables

**Table:** 

AA	Alzheimer’s Association
AD	Alzheimer’s Disease
ADL	Activities of Daily Living
BPSD	Behavioral and psychological symptoms of dementia
BPST	Brief psychosocial therapy
CBT	Cognitive behavioral therapy
CI	Confidence interval
CSF	Cerebral spinal fluid
DSM-V	Diagnostic and statistical manual of mental disorders fifth edition
GI	Gastrointestinal
IADLs	Instrumental activities of daily living
ISTAART	International Society to Advance Alzheimer’s Research & Treatment
LBD	Lewy body dementia
MAOI	Monoamine oxidase inhibitors
MMSE	Mini-Mental State Examination
MoCA	Montreal cognitive assessment
NMDA	N-methyl-D-aspartate
NPI	Neuropsychiatric inventory
NPS	Neuropsychiatric symptoms
PET	Positron emission tomography
SPECT	Single-photon emission computerized tomography
SSRIs	Selective serotonin reuptake inhibitors
SNRIs	Serotonin and Norepinephrine Reuptake Inhibitors
